# Multimodal prehabilitation in colorectal cancer patients to improve functional capacity and reduce postoperative complications: the first international randomized controlled trial for multimodal prehabilitation

**DOI:** 10.1186/s12885-018-5232-6

**Published:** 2019-01-22

**Authors:** Stefanus van Rooijen, Francesco Carli, Susanne Dalton, Gwendolyn Thomas, Rasmus Bojesen, Morgan Le Guen, Nicolas Barizien, Rashami Awasthi, Enrico Minnella, Sandra Beijer, Graciela Martínez-Palli, Rianne van Lieshout, Ismayil Gögenur, Carlo Feo, Christoffer Johansen, Celena Scheede-Bergdahl, Rudi Roumen, Goof Schep, Gerrit Slooter

**Affiliations:** 1 Department of Surgical Oncology, Máxima Medical Center, Veldhoven, the Netherlands; 2 Department of Anesthesiology, the Montréal General Hospital, McGill University, Montréal, Canada; 3 Danish Cancer Society Research Center, Copenhagen, Denmark; 4 Department of Surgery, Center for Surgical Science, Zealand University Hospital, Køge, Denmark; 5 Department of Anesthesiology, Foch Hôpital, Paris, France; 6 Department of Sports Medicine, Foch Hôpital, Paris, France; 7 Netherlands Comprehensive Cancer Organisation, Utrecht, the Netherlands; 8 Department of Anesthesiology, Hospital Clinic de Barcelona, IDIBAPS, University of Barcelona, Barcelona, Spain; 9 Department of Nutrition, Máxima Medical Center, Veldhoven, the Netherlands; 10 Department of Surgery, S. Anna University Hospital, Ferrara, Italy; 11 Department of Oncology, Finsen Center, Rigshospitalet, Copenhagen, Denmark; 12 Department of Kinesiology and Physical Education, McGill University, Montréal, Canada; 13 Department of Sports Medicine, Máxima Medical Center, Veldhoven, the Netherlands; 14 Department of Surgery, Máxima Medical Center, P.O. Box 7777, Veldhoven, the Netherlands

**Keywords:** Prehabilitation, Colorectal surgery, Functional capacity, Enhanced recovery after surgery, Comprehensive complication index, Postoperative complications, Colorectal cancer

## Abstract

**Background:**

Colorectal cancer (CRC) is the second most prevalent type of cancer in the world. Surgery is the only curative option. However, postoperative complications occur in up to 50% of patients and are associated with higher morbidity and mortality rates, lower health related quality of life (HRQoL) and increased expenditure in health care. The number and severity of complications are closely related to preoperative functional capacity, nutritional state, psychological state, and smoking behavior. Traditional approaches have targeted the *postoperative* period for rehabilitation and lifestyle changes. However, recent evidence shows that the *preoperative* period might be the optimal moment for intervention. This study will determine the impact of multimodal prehabilitation on patients’ functional capacity and postoperative complications.

**Methods/design:**

This international multicenter, prospective, randomized controlled trial will include 714 patients undergoing colorectal surgery for cancer. Patients will be allocated to the intervention group, which will receive 4 weeks of prehabilitation (group 1, prehab), or the control group, which will receive no prehabilitation (group 2, no prehab). Both groups will receive perioperative care in accordance with the enhanced recovery after surgery (ERAS) guidelines. The primary outcomes for measurement will be functional capacity (as assessed using the six-minute walk test (6MWT)) and postoperative status determined with the Comprehensive Complication Index (CCI). Secondary outcomes will include HRQoL, length of hospital stay (LOS) and a cost-effectiveness analysis.

**Discussion:**

Multimodal prehabilitation is expected to enhance patients’ functional capacity and to reduce postoperative complications. It may therefore result in increased survival and improved HRQoL. This is the first international multicenter study investigating multimodal prehabilitation for patients undergoing colorectal surgery for cancer.

**Trial registration:**

Trial Registry: NTR5947 – date of registration: 1 August 2016.

## Background

Colorectal cancer (CRC) is the second most prevalent type of cancer in the world, with over 1.4 million cases and 693.900 deaths a year [1]. The only way to cure this condition is by surgical removal of the tumor. However, postoperative complications occur in up to 50% of patients and they are associated with higher morbidity and mortality rates, increased expenditure on health care and poorer health related quality of life (HRQoL) [2–4]. Major surgery brings a 20 to 40% reduction in physiological and functional capacity, even in absence of complications [5]. This diminished reserve increases the level of fatigue up to months after hospital discharge [6]. Only 40% of patients return to their preoperative baseline functional capacity (as measured by VO2 peak). Moreover, a proportion of colorectal cancer patients are eligible for (neo)adjuvant which is known to further compromise functional recovery and survival [7, 8].

There is emerging evidence suggesting that many of the negative effects of major surgery can be reduced through the attenuation of surgical stress [9]. Efforts to improve the recovery process have primarily focused on the intraoperative factors (such as minimally invasive surgery and afferent neural blockade [10]) and postoperative interventions (examples being “fast track” early nutrition and mobilization [11]). The latter protocols have been designed to facilitate the return of functional activities and accelerate convalescence. However, the *postoperative* period may not be the best time to ask surgical patients to make significant changes in their nutrition and exercise since patients are tired and concerned about perturbing the healing process. As well as anxious about possible additional treatments for their underlying condition. The *preoperative* period may in fact be a better time to intervene in the factors that contribute to recovery, both physical and mental, and alleviate some of the emotional distress associated with the anticipation of surgery and the recovery process [12–14].

The process of improving the functional capacity of the individual in order to enable them to withstand an incoming stressor has been termed *prehabilitation* [15, 16]. Although some approaches have focused on education to prepare patients for procedures [17], few steps have been taken to systematically enhance functional capacity before surgery. With most studies performing single modal interventions only. To investigate the impact of preoperative exercise on the recovery of functional capacity after colorectal surgery, a few pilot studies have been performed which show promising results [14–16, 18, 19].

Subgroup analysis of the study of Carli et al., showed that patients whose functional exercise capacity improved preoperatively, recovered relatively well in the postoperative period - regardless of the exercise technique [20]. However, one-third of patients deteriorated preoperatively despite the exercise regimen, and these patients were also at greater risk of prolonged recovery after surgery. Poor preoperative physical function (fatigue, malnutrition and physical performance) and the presence of anxiety and depression were also significant confounding predictors of prolonged recovery [21–25]. These results suggest that exercise training alone is not sufficient to attenuate the stress response in all patients and that it is also important to address factors such as nutrition and coping behavior that promote beneficial adaptation to training. Gillis et al. conducted a pilot study that showed that significant changes in postoperative functional exercise capacity can be achieved with a prehabilitation program [26]. However, they did not address the clinically relevant relationship between preoperative functional capacity (an increase of more than 20 m on the six-minute walk test (6MWT)), and the postoperative outcome (comprehensive complication index (CCI) reduction of 30%). If functional capacity can be improved preoperatively, we may expect a reduction in postoperative complications.

Since it has been established that the number and severity of complications are closely related to preoperative functional capacity, nutritional status, smoking behavior and psychological well-being, there has been increasing interest in targeting these issues with a multimodal intervention program [15]. From a physiological point of view and based on limited practical experience, it seems feasible to achieve clinically relevant effects during the period of 4–5 weeks between diagnosis and operation [14, 15, 27]. However, this can only be achieved with targeted interventions that include exercise, nutrition, stopping smoking, and psychological support.

### Study objectives

The general aim of this study is to investigate whether multimodal prehabilitation could enhance postoperative outcome using the CCI and 6MWT. Secondary outcomes will include patient reported outcome measures (PROMs) such as HRQoL and depression and anxiety scores, functional capacity measurements, nutritional and smoking status, length of hospital stay, study compliance, patients’ satisfaction and a cost-effectiveness analysis.

## Methods

This is an international multicenter, randomized controlled trial with two study groups. Written informed consent will be obtained from all patients. The trial will be conducted according to the rules of Good Clinical Practice and a Data Safety Monitoring Board (DSMB) has been appointed to monitor (serious) adverse events. The Netherlands Comprehensive Cancer Organisation will be responsible for quality control and data management. Ethical approval for this study was granted by the Medical Ethics Committee of the Máxima Medical Center (Veldhoven, the Netherlands) under reference number W16.100/NL58281.015.16. Important protocol modifications will be addressed to relevant parties.

### Study population

Adult patients (> 18 years) undergoing elective colorectal resection for cancer are eligible for inclusion. We will include 714 patients: 357 in each arm. We expect a dropout rate of 10% based on previous pilot studies. The estimated duration of the recruitment period is two years. Exclusion criteria are metastatic disease known preoperatively, paralysis or patients with mobility problems (who are unable to exercise), premorbid conditions or orthopedic impairments that contraindicated exercise, cognitive disabilities, chronic renal failure (dialysis or creatinine > 250 mmol), ASA score 4 or higher, and illiteracy (inability to read and understand the language of the country where the study will be performed).

### Participating centers

Patients from the Máxima Medical Center (coordinating hospital, Eindhoven-Veldhoven, the Netherlands), the Montréal General Hospital (Montréal, McGill, Canada), Zealand University Hospital (Zealand Region, Denmark), Foch Hôpital (Paris, France), the Saint Anna University Hospital of Ferrara (Ferrara, Italy), and Hospital Clinic de Barcelona (Barcelona, Spain), will be included in this study.

### Randomization

Patients will be block randomized with a 1:1 allocation by means of randomization software (Research Manager clinical trial data management system, Deventer, the Netherlands), stratified by study sites, tumor location and neoadjuvant treatment. Patients will be allocated either to the intervention group, which will receive 4 weeks of prehabilitation, or to the control group, which will receive no prehabilitation.

**Fig. 1:**
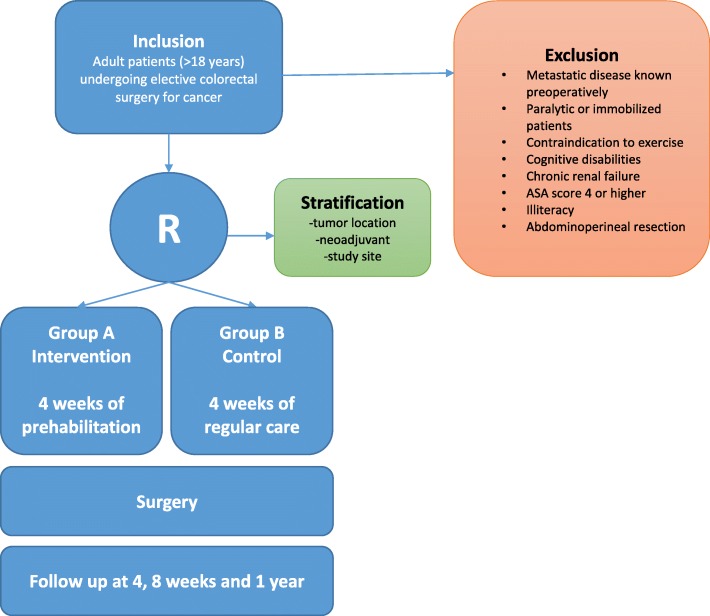
Flow diagram for study participants

In all participating centers, both the investigator and the surgeon responsible will verify eligibility. If the indication for surgery is established, patients will be screened by the medical research team for health conditions that prohibit participation in the program. They will then be called by the research investigator and an appointment will be made to provide written and oral information about the trial during a scheduled outpatient appointment. Patients will be given enough time to enquire about the details of the trial and to decide whether or not they wish to participate. Patients will be required to sign the informed consent form in the presence of the surgeon or investigator. A participant flow diagram is shown in Fig. 1. After the study has been explained and consent is obtained, there will be a multidisciplinary assessment. Based on intake by the sports physician, the physiotherapist/kinesiologist, the nutritionist and the case manager/psychologist, an individual prehabilitation program will be started during four weeks in the intervention group.

### Study outline

Routine standard preoperative and postoperative clinical care in the participating institutions do not currently include special nutrition, exercise and psychological support to cope with anxiety before surgery. As usual, patients are evaluated in the preoperative clinic before surgery to determine whether they are fit for surgery and to adjust their medication for co-morbid conditions. It is not common practice after surgery to offer them an in-hospital exercise program but patients are given generic instructions by the surgeons about mobilization and returning to normal activities. Some patients may be referred to a physiotherapist by their surgeon. Nutrition, exercise and coping strategies will be introduced solely for research purposes. All patients will be screened four weeks before surgery to capture insufficient hemoglobin levels (thresholds in Canada: > 11.2 g/dl, Europe: > 7 mmol/l). Hemoglobin levels in patients with iron insufficiency will be optimized using iron injections (ferinject).

Perioperative care will be based on a standardized, multi-element, evidence-based, comprehensive, ERAS guideline in line with the consensus review of optimal care for patients undergoing colorectal surgery [27]. The guideline will be applied in all participating centers to improve generalizability.

The diagnostic work-up for patients with a tumor suspected for malignancy at colonoscopy will be finalized within one week while awaiting definitive pathology. An individual treatment strategy will then be proposed by the multidisciplinary team. Patients that meet the criteria for the trial will be scheduled approximately five weeks after the final diagnosis. This schedule allows for the implementation of a four-week prehabilitation program.

The multidisciplinary multimodal prehabilitation program is composed of 4 elements: exercise training, nutritional intervention, smoking cessation and psychological support. The exact interventions are shown in Table 1 and described in detail below:

**Table 1:** Prehabilitation interventions

Prehabilitation randomized controlled trial scheme							
	Preoperative			Operation	Postoperative		
Weeks	−5	−4	−1	0	4	8	52
	Before start	Baseline (t0)	Preoperative (t1)	Surgery	30 day follow up (t2)	8 weeks follow up (t3)	1 year follow up (t4)
Gastroenterologist	Inform patient	–	–	–	–	–	–
Casemanager	Inclusion patient	G8 score	–	–	–	–	–
Sport physician	–	Informed consent VO2max Anaerobic threshold Exercise-ECG	VO2max Anaerobic threshold Exercise-ECG	–	VO2max Anaerobic threshold Exercise-ECG	–	–
Physiotherapist	–	6MWT Stair climb test Sit-to-stand test 1-RM Activity questionnaire Fried Frailty Score	6MWT Stair climb test Sit-to-stand test Activity questionnaire	–	6MWT Stair climb test Sit-to-stand test Activity questionnaire	6MWT Stair climb test Sit-to-stand test Activity questionnaire	–
Dietician	Food diary	Height, weight Weight loss %^a^ Anthropometry Hand grip strength PG-SGA	Food diary Height, weight Weight loss %^a^ Anthropometry Hand grip strength PG-SGA	–	Height, weight Weight loss %^a^ Hand grip strength PG-SGA	Height, weight Weight loss %^a^ Wrist/abdominal/ Hand grip strength PG-SGA	–
Psychologist	–	Intake Coping with anxiety^c^	–	–	–	–	–
Anesthesiologist	–	–	Preoperative screening^b^	ERAS^b^	–	–	–
Surgeon	–	–	ERAS**	–	–	–	–
Surgical resident	–	–	–	–	Outpatient data	–	–
Researcher	–	HRQoL GAD-7 PHQ-9	HRQoL GAD-7 PHQ-9	–	30-day morbidity and mortality HRQoL GAD-7 PHQ-9	HRQoL GAD-7 PHQ-9	Mortality HRQoL GAD-7 PHQ-9

^a^weight loss in the past 3–6 months. ^b^Following Enhanced Recovery After Surgery (ERAS) guidelines. ^c^when indicated, as stated in the protocol

#### Exercise program

An exercise specialist (kinesiologist, sport physician) will assess the patients’ mobility and his/her capacity to exercise using a cardiopulmonary exercise test (CPET). The CPET values will be used to establish an individualized dosing of training embedded in a standardized training program [28].

- The interval training duration is 28–32 min and performed with 4 min of warm-up at moderate intensity, 4 intervals of High intensity (2–3 min) and 4 intervals of Moderate intensity (4 min).
- The workload is dosed at the wattage corresponding to 90% the peak wattage as attained by the CPET. This is aimed to result in a metabolic response ranging from 85 to 100% of VO2peak during the high intensity intervals. This corresponds with a heart-rate of 85–100% of maximal heart rate, and a Borg score of 15–17. Moderate intensity is set at 30% of peak wattage of the CPET test. This is aimed to have recovery at a level around or just below Aerobic Threshold.
- If the patient is unable to complete the high-intensity bout for 4 periods of 2 min (=less than 8 min’ exercise in the high intensity range), the intensity is reduced by 10 %. The intensity is reduced further – in steps of 10 % – until the patient can complete the 4 bouts of at least 2 min. If the patient is able to complete all 4 high intensity bouts for 3 min (12 min in the high intensity range) the load is increased with 10%. If necessary fine-tuning can be done using steps of 5%.

In addition to the preoperative exercise program, patients will receive information about breathing techniques to prevent pneumonia.

Patients in the intervention group will have three supervised in-hospital training sessions per week during four weeks. This includes interval training and resistance training.

The interval training duration is 28–32 min and performed with 4 min of warm-up at moderate intensity, 4 intervals of High intensity (2–3 min) and 4 intervals of Moderate intensity (4 min).

The workload is dosed at the wattage corresponding to 90% the peak wattage as attained by the CPET. This is aimed to result in a metabolic response ranging from 85 to 100% of VO2peak during the high intensity intervals. This corresponds with a heart-rate of 85–100% of maximal heart rate, and a Borg score of 15–17 Moderate intensity is set at 30% of peak wattage of the CPET test. This is aimed to have recovery at a level around or just below Aerobic Threshold.

If the patient is unable to complete the high-intensity bout for 4 periods of 2 min (=less than 8 min’ exercise in the high intensity range), the intensity is reduced by 10 %. The intensity is reduced further – in steps of 10 % – until the patient can complete the 4 bouts of at least 2 min. If the patient is able to complete all 4 high intensity bouts for 3 min (12 min in the high intensity range) the load is increased with 10%. If necessary fine-tuning can be done using steps of 5%.

Resistance training is composed as 2 series of 10 repetitions of 6 exercises targeting all major muscle groups).

- The strength training consists of two series of 10 repetitions of six exercises: leg press, chest press, abdominal crunches floor, lat pull down, low row and step up.
  - ○ In week 1 using 65% of calculated 1RM (baseline)
  - ○ In week 2 using 70% of calculated 1RM (baseline)
  - ○ In week 3 using 65% of calculated 1RM (at 3 weeks).
  - ○ In week 4 using 70% of calculated 1RM (at 3 weeks)
- The 1RM will be determined at baseline and at 3 weeks using. The strength exercises are performed according to: 2 s of concentric strength and 2 s of eccentric strength. The 1RM is calculated by the Brzycki formula:
  - ○ 1RM = W*36/(37 –r)
    *W* = weight in kilograms
    *r* = repetitions
- The last bout with 10 repetitions needs to be attainable. If this is not the case in the next session dosing will be 5–10% lower. If in the last bout it appears that exercises are to low (≥15 repetitions will be achieved), in the next sessions dosing will be 5–10% higher.

Patients will also be given instructions about how to conduct aerobic exercises at home. Patients are instructed to aim at 60 min walking and/or cycling a day, but at least 30 min a day. If possible, patients can do more than 60 min of walking or cycling every day. In case of a low exercise capacity it is advised to walk/cycle 2–3 times a day for periods of 10–20 min. Also an electric bicycle, a stationary bicycle and/or a walking aid (walker) is allowed if necessary.

Patients in both the intervention and control group will wear an accelerometer for four weeks to count the number of steps walked in order to record the overall activity.

The conventional wisdom is that training blocks of 3–5 min are particularly effective in terms of enhancing exercise capacity. Most of the nine studies that produced the largest increases in VO_2_max (∼0.85/min) used blocks of 3–5 min and HIT. Many of these studies presented either individual data or ranges for VO_2_max values pre- and post-training, and an appraisal of these data suggests that a marked training response was seen in all subjects [29].

In the intervention group we expect to achieve the following improvements after four weeks of prehabilitation compared to baseline measurements: a 10% increase in VO2 peak, a 15% increase in VO2 at anaerobic level, a 20–40% increase in 1-RM tests and an increase of > 20 m in the 6MWT [12, 15, 16, 19, 27].

#### Nutritional assessment and intervention

The nutritionist will complete nutritional assessments at baseline appointments and during the prehabilitation program using the patient-generated subjective global assessment (PG-SGA), body composition (skinfold measurements, mid upper-arm muscle area), hand grip strength and nutritional intake (caloric and protein intake), and a patients’ three-day food diary.

We aim to establish an anabolic condition preoperatively. In cachectic and sarcopenic patients we try to increase lean body mass by 1–2 kg or more during four weeks of prehabilitation. In order to achieve this goal, the target dietary protein intake will be 1.5–1.8 g/kg body weight in all patients [16, 30–35].

Participants will receive high-quality protein supplements containing 30 g of whey protein following exercise and before sleep. Dietary advice will be given in order to achieve adequate oral protein intake spread properly across meals. Since vitamin D is associated with muscle mass and muscle strength [36, 37], vitamin D will be supplemented daily according to guidelines of the Health Council of the Netherlands (10 μg for women aged 50-69y, for men <70y and women <50y with colored skin and/or little sun exposure and 20 μg for women and men aged 70 y or older) [38]. Besides vitamin D, many elderly patients may have other micronutrient deficiencies or ingest vitamins and minerals below recommended doses before and after surgery. Therefore, all other vitamins and minerals are supplied in a multivitamin/mineral supplement containing 50% of the recommended daily allowance.

During the period of hospitalization, the time (in days) that patients consume nil per mouth is recorded. Also, on the day of discharge, a trained dietician performs a 24-h recall questionnaire to estimate oral protein- and energy intake. Nutritional status assessment (PG-SGA) will be performed at 4 and 8 weeks post-surgery by an investigator – trained by a registered dietician.

#### Smoking cessation

A smoking cessation program with intensive counseling and nicotine replacement therapy (NRT) will be offered to all patients during the weeks of prehabilitation. Past and current smoking is measured using questionnaires. Counselling includes group weekly group sessions and telephone calls. NRT may consist of any type of replacement therapy. Approximately 15–20% of our patients are smokers when cancer is diagnosed [13]. The goal is to achieve a smoking cessation rate of 80% before surgery.

#### Psychological coping

It is expected that patients undergoing surgery for cancer are anxious with some component of depression. Since both anxiety and depression can influence the motivation to carry out social and functional activities, psychological strategies can be put in place to help patients to cope with the stress of surgery and disease. Therefore, patients will be screened for anxiety and depression using the GAD-7 and PHQ-9 questionnaires. If these questionnaires result in a high score (GAD-7 of 10 or higher; PHQ-9 score 15 or higher), patients are considered high-risk and will be offered a referral to a psychologist. Referred patients will receive a total of 1.5 h of psychological intervention in the first session and more sessions during the 4 weeks of prehabilitation if necessary.

All patients in the intervention group will be given instructions on relaxation and breathing techniques by a trained investigator. They will be given an instruction CD, which they can use for relaxation techniques at home. After the program, patients will be asked if their perceived usefulness of these techniques. For psychological support, the intervention group will be contacted weekly by the investigator by phone. During these 5–15 min phone calls, a researcher will shortly evaluate personal progression by a standardized set of questions. In order to enhance adherence to the prehabilitation program, all patients in the intervention group will receive an instructional brochure that includes information about all elements of the program.

### Study outcomes

The initial primary outcome will be postoperative complications, as scored by the Comprehensive Complication Index [39], with the relevant data being collected as a continuous variable and calculated using the sum of morbidity and mortality presented on the Clavien Dindo classification [40]. The CCI score will be calculated at the 30 days of follow-up. The second primary outcome will be the 6MWT measured at 4 weeks after surgery and compared to baseline. The 6MWT will additionally be measured directly after prehabilitation and 8 weeks after surgery.

Secondary outcomes will include patient reported outcome measurements (PROMs) such as health related quality of life (HRQoL) (EORTC QLQ-CR29 and EORTC QLQ-C30 and RAND questionnaires) and depression and anxiety scores (GAD-7, PHQ-9 questionnaires), functional capacity (CPET including VO2max, VO2peak, AT, the sit to stand test, stair climb test, hand grip strength and activity questionnaire), nutritional status (3-day food diary, PG-SGA, anthropometry), postoperative complications, length of hospital stay, study compliance, patient satisfaction and a cost-effectiveness analysis. All secondary outcomes are measured at baseline, the week before surgery, 4 and 8 weeks and 1 year post-surgery.

### Statistical analysis

Baseline characteristics of both groups will be compared to assess the adequacy of the randomization. Data will be analyzed on an intention-to-treat basis. In addition, a per-protocol analysis will be performed. Trial results will be published in a respective journal. Primary and secondary outcomes for the intervention and control groups will be compared.

The primary outcome CCI will be described as the mean plus the standard deviation (SD). Since we expect CCI to be right skewed we will also describe CCI as the median plus interquartile range (IQR) and percentage above 20. To test the hypothesis (H0) that the study arms result in similar CCIs (in other words, prehabilitation does not prevent postoperative complications), we will use the Student’s T-test if data are normally distributed. Mann-Whitney U if data are not normally distributed or statistical methods that take into account the possible zero-inflated nature of the data. The second primary outcome 6MWT is a continuous variable. This data will be stated as means, plus SD, at each time point. To accommodate the repeat measurements for individuals, we will use a generalized linear mixed model to statistically test the hypothesis of both study arms being equal in terms of functional capacity over time.

All secondary outcomes will be described as means plus SD or median plus IQR, with the data being continuous, and measures for each time point being normally and non-normally distributed respectively. Categorical parameters will be described as number plus percentage per time point. Statistical methods will include t-test and the Mann-Whitney U test for continuous parameters, distributed either normally or not normally respectively, at a single postoperative time-point. Categorical outcomes will be analyzed with Chi-square testing or regression analysis (logistic, ordinal or nominal, depending on the definition of the parameter) for single time points. The size of the sample will be calculated on the basis of the primary aim: the reduction of postoperative complications as determined with the CCI score. With our population variables, the CCI mean is 10.4 (SD 14), and the target reduction is 30%. We use an alpha of 0.05 and power of 0.80 (two-sided test). We expect a dropout rate of 10%. We therefore need 714 patients: 357 in each arm. This gives us sufficient statistical power to demonstrate the expected proportion of difference (55% versus 20%) in the 6MWT between baseline and surgery based on previous studies [14, 27]. Approximately 600 eligible CRC patients undergo surgery in one of the six participating hospitals annually. This implies that we will complete inclusion within two years with the inclusion period being followed by a year of follow-up.

Due to limited clinical data regarding effect size of the primary endpoint – CCI - an interim analysis will be performed. The interim analysis is planned if half of the intended number of subjects have completed the 4 week assessment (i.e. timing of the primary endpoint assessment). The intention of the interim analysis is to terminate the study if there is a statistical significant difference between study arms.

### Economic evaluation

To analyze cost-effectiveness, we will focus on the results of the program defined as reduction of complications, improving survival, less need for postoperative care and improvements in social productivity. The economic evaluation will be performed per participating center and includes incremental cost-effectiveness and cost-utility analysis using the RAND and iMTA-PCQ questionnaires. The cost-effectiveness ratio will be calculated by dividing the difference between the mean total costs for the exercise and control groups by the difference in the mean effect in the groups [41]. The cost-utility ratio expresses the additional costs of the intervention compared with the control group per quality-adjusted life years [42].

## Discussion

Despite advances in surgical techniques and improvements in postoperative care, morbidity and mortality remain high in CRC patients undergoing surgery. Postoperative complications occur in up to 50% of patients and surgery is associated with a 20 to 40% reduction in physiological and functional capacity [2–5].

Since we know that the number and severity of complications are, or may be, associated with preoperative functional capacity, nutritional status, smoking behavior and psychological well-being, it is incumbent on us to test a multimodal intervention program that targets these issues. Traditional approaches have focused on the postoperative period for rehabilitation and lifestyle changes. However, recent evidence has shown that the preoperative period is a better time to intervene [27]. Therefore, we initiated the first international randomized controlled trial on multimodal prehabilitation for patients undergoing colorectal surgery for cancer.

Patient lifestyle (stated as inactivity, obesity, dietary pattern, and smoking behavior) is an important contributor to the development of CRC [43]. Moreover, CRC patients often develop problems with their nutritional status which may aggravate deconditioning and muscle wasting (sarcopenia) [31, 32, 44]. This implies that, particularly in this group of patients, there is considerable potential for improvement in both nutritional status and functional exercise capacity [45–47]. A recent review indicated that optimizing functional exercise capacity in the surgical population can, by comparison with controls, result in fewer postoperative complications, shorten the length of hospital stay, reduce disability, and improve quality of life [48]. However, there have been no previous studies rigorously evaluating the impact of multimodal prehabilitation prior to digestive surgery.

Interventions that involve physical exercise training for endurance and strength, nutrition, mental support and smoking behavior have all found an independent and clinically relevant effect on the reduction of postoperative complications in small studies. If all these interventions are orchestrated in an innovative prehabilitation program, it may prove feasible to design a highly effective and comprehensive intervention. Synergy may result from these individual interventions if they are applied in a multimodal program since it is known that protein supplements one hour after exercise improve uptake and enhance anabolic effects [30–33, 44, 49]. Nutritional supplementation four weeks before and after surgery has been shown to enhance preoperative functional walking capacity and recovery in patients undergoing colorectal resection for cancer [34]. Moreover, the release of dopamine during exercise improves the psychological mindset and smoking cessation improves the ability to perform exercise.

After the diagnosis of CRC, there is a relatively short period of 4–5 weeks before the actual surgery. A rigorous intervention program of prehabilitation is therefore required that is closely coordinated with the entire medical treatment program. An additional potential benefit is the empowerment of patients, who may then play an active role in coping with their disease. From a physiological point of view and based on limited practical experience, it seems feasible to achieve clinical relevant effects during the period of four weeks between diagnosis and surgery. However, this is possible only with a combination of robust innovative interventions involving nutrition, physiological support, smoking cessation and exercise training. These ideas are supported by Carli et al., who stated that a multidisciplinary prehabilitation program needs to be developed, tested, implemented and delivered to patients [16, 35].

Another reason why optimal recovery after surgery is important is because it will increase the potential of patients to withstand additional therapies such as chemotherapy, targeted immunotherapy, metastatic disease resection and/or hyperthermic intraperitoneal chemotherapy (HIPEC) [50, 51]. A study by the Dutch cancer register included 11.000 stage-3 CRC patients (2008–2013) [7]. 4899 of whom were not treated with chemotherapy. The five-year survival rate in this group was only 39%. If chemotherapy started > 12 weeks postoperatively, the five-year survival rate increased to 54%. When chemotherapy began < 6 weeks after the operation, this rate increased further to 76%. Improved functional capacity may facilitate an earlier start of adjuvant chemotherapy and thereby increasing survival an improving HRQoL [7, 8, 52, 53].

Despite previous evidence from small-scale trials, there are currently no standardized prehabilitation programs and they are therefore not mentioned in current medical guidelines. This highlights the need to design, test, optimize, and implement a multimodal program for maximizing improvements in nutritional status and functional capacity prior to surgery. We expect to see a reduction in postoperative complications, Length Of hospital Stay (LOS), intensive care stay, 30-day mortality rate, and health expenditure due to the multimodal prehabilitation program. The sum of all these separate outcomes will be measured using the Comprehensive Complication Index (CCI), which is a relatively new and interesting outcome measure [40]. As stated in the literature, patients with postoperative complications report a lower HRQoL than patients without. We will be the first to determine whether the HRQoL of patients with postoperative complications is inferior both preoperatively and 1 year postoperatively than the HRQoL of patients without complications. We expect poorer HRQoL in patients with complications preoperatively and one year postoperatively than in patients without complications.

Limitations of the study should be noted as well. Due to its nature this study is performed non-blinded. This may result in higher dropout rates or an increased activity level in the control group, due to the growing and intuitive understanding of the benefits of exercise training and optimal nutrition. To limit this potential bias of increased activity, we will introduce activity trackers to all patients. We do realize patient characteristics, social circumstances and healthcare facilities will not be the same in all countries. Therefore, we will stratify per participating center. Our international approach of the randomized controlled trial will demonstrate that a worldwide implementation may be possible. In case our multimodal program proves to enhance postoperative outcome, it will be impossible to discover which element of the program attributed most. A large sample size and different outcome measurement will facilitate subgroup analyses, to determine effects of the program within different areas, such as functional capacity, body composition, and quality of life. Also, to allow for comparison of the effects of single modal interventions in this multimodal program. Our large sample size will also give us the possibility to analyze the possible effect differences of an intensive hospital-based prehabilitation program in different patient groups. This way, prehabilitation in the current form can be implemented for patient groups who will benefit the most from intensive hospital-based training. For less high-risk patients, future research could focus on programs in which training is less intensively monitored, such as home-based programs.

Our study investigating prehabilitation is a good example of research in prevention and it will be the first to systematically implement existing knowledge from a variety of different medical specialties and basic science into a four-element multimodal preoperative program for CRC patients with the aim of improving functional capacity and reducing the postoperative complication rate.

## Abbreviations

6-MWT: Six minute walk test
CAL: Colorectal anastomotic leakage
CCI: Comprehensive complication index
CHAMPS: Community health activities model program for seniors
CPET: CardioPulmonary exercise testing
CRC: ColoRectal cancer
DSMB: Data safety monitoring board
ERAS: Enhanced recovery after surgery
GAD-7: Generalized anxiety disorder 7
HIPEC: Hyperthermic intraPEritoneal chemotherapy
HIT: High intensity training
HRQoL: Health related quality of life
LOS: Length of hospital stay
MMC: Máxima medical center
NRT: Nicotine replacement therapy
PG-SGA: Patient generated subjective global assessment
PHQ-9: Patient health questionnaire 9
SD: Standard deviation
